# The Association Between Usage of Colchicine and Pneumonia: A Nationwide, Population-Based Cohort Study

**DOI:** 10.3389/fphar.2019.00908

**Published:** 2019-08-16

**Authors:** Tsung-Lin Tsai, James Cheng-Chung Wei, Yue-Ting Wu, Yueh-Han Ku, Kun-Lin Lu, Yu-Hsun Wang, Jeng-Yuan Chiou

**Affiliations:** ^1^Institute of Medicine, Chung Shan Medical University, Taichung, Taiwan; ^2^Department of Medicine, Chung Shan Medical University Hospital, Taichung, Taiwan; ^3^Graduate Institute of Integrated Medicine, China Medical University, Taichung, Taiwan; ^4^Department of Medical Research, Chung Shan Medical University Hospital, Taichung, Taiwan; ^5^School of Health Policy and Management, Chung Shan Medical University, Taichung, Taiwan

**Keywords:** gout, pneumonia, colchicine, cohort, population-based study

## Abstract

**Objectives:** A previous study suggested that colchicine may cause leukopenia and increase the risk of infection, such as pneumonia. Thus, we investigated the potential relationship between colchicine use and risk of developing pneumonia.

**Methods:** Data were collected from Taiwan’s National Health Insurance Research Database (NHIRD), a nationwide, population-based database. A 13-year retrospective cohort study was conducted, and all investigated subjects were identified by International Classification of Disease, Ninth Revision, Clinical Modification, codes between 2000 and 2012. Propensity score matching was applied to adjust for potential confounding variables, and then Cox proportional hazard model was used to evaluate the hazard ratio (HR) of pneumonia in gout patients and its associations with colchicine use, colchicine dosage, and days of colchicine use.

**Results:** A total of 24,410 gout patients were enrolled in this study, including 12,205 cases who were treated with colchicine (colchicine group) and 12,205 cases who did not receive colchicine (non-colchicine group). The overall incidence rates of pneumonia in the colchicine group and non-colchicine group were 18.6 and 12.6 per 1,000 person-years, respectively. The colchicine group had a higher risk of pneumonia as compared with the non-colchicine group [adjusted HR, 1.42; 95% confidence interval (CI), 1.32 to 1.53; P < 0.05]. High cumulative dose and days of colchicine use notably increased the risk of contracting pneumonia.

**Conclusion:** This nationwide population-based cohort study reveals that gout patients taking colchicine are at increased risk of developing pneumonia compared with gout patients who do not use colchicine. Therefore, it is crucial that gout patients being treated with colchicine be given the minimally effective dosage for the shortest possible duration to minimize their risk of pneumonia.

## Introduction

Gout is the most common form of inflammatory arthritis and has a considerable deleterious impact on daily life (Roddy and Choi, 2014; Kuo et al., 2015b). According to a nationwide population study, the prevalence of gout in Taiwan was reported to be 6.24% in 2010 (Kuo et al., 2015a). Colchicine is an anti-inflammatory drug that is effective for treating and preventing gouty arthritis (Hainer et al., 2014).

Recent studies have suggested that colchicine may inhibit neutrophil function (Asako et al., 1992; Cronstein et al., 1995; Chia et al., 2008). It has also been reported that colchicine may exert an immuno-suppressive effect (Dalbeth et al., 2014). Moreover, *in vivo* research on myocarditis revealed that colchicine may not be suitable for treating patients with viral myocarditis because it can exacerbate the severity of viral infection in both the heart and the pancreas (Smilde et al., 2016). Moreover, an early case report showed that neutropenia may occur in patients using the recommended colchicine dosage (Dixon and Wall, 2001). Another case report showed that a patient with colchicine administration developed leukopenia, a disease that renders patients prone to infectious disease, such as pneumonia (Beggs et al., 2012). Furthermore, a cohort study showed that infections in gout patients may be attributable to colchicine (Spaetgens et al., 2017).

Pneumonia is the most serious infectious disease of the respiratory system and was the third highest cause of mortality in Taiwan in 2017 (Shen et al., 2016). Since colchicine might impair normal immunity, it is crucial to gain a better understanding of the relationship between colchicine and pneumonia using a long-term population-based database (Spaetgens et al., 2017).

## Methods

### Database

Taiwan’s National Health Insurance Research Database (NHIRD) was established in 1995, and currently contains comprehensive health care data for almost all Taiwanese citizens. The database is composed of all National Health Insurance (NHI) claims data, and includes information on hospitalization, emergency care, and medical visits. Taiwan’s NHI program had a coverage rate >99% in 2010, and thus the NHIRD contains data for approximately 23 million beneficiaries (Hsing and Ioannidis, 2015; Su et al., 2018). In Taiwan, 93% of medical institutes were contracted by the NHI. The NHIRD releases anonymized data for medical research and is one of the world’s largest medical databases of its kind.

The Longitudinal Health Insurance Research Dataset 2000 (LHIRD 2000) contains the clinical information of 1 million beneficiaries randomly selected from the NHIRD during the period 2000 to 2013 (Chen et al., 2018). Moreover, the diagnosis in the LHIRD is made by physicians using the International Statistical Classification of Diseases and Related Health Problems, 9th Revision, Clinical Modification (ICD-9-CM). In Taiwan, patients’ medical data include drug items and their corresponding NHI code, dosage, frequency of use, and number of days prescribed. This information can be used for detecting drug interactions and potential duplicate medications when patients visit multiple hospitals. Every NHI code corresponds to a code in the five-level ATC classification system recommended by the World Health Organization (WHO) for studies on drug utilization (Hsu et al., 2011).

### Study Design

This population-based nationwide retrospective cohort study analyzed data from Taiwan’s National Health Insurance Research Database from 2000 to 2013. The study was approved by the institutional review board of Chung Shan Medical University Hospital, with IRB number CS17114.

### Patients Selection

We identified 1 million people from the database. First, we selected patients 20 years or older with newly diagnosed gout, based on the ICD-9-CM code 274, from 2000 to 2012. To ensure disease code accuracy, we selected patients whose clinical history included at least three outpatient visits or one hospitalization. Second, individuals were divided into two groups.

The colchicine group comprised individuals who had used colchicine within 1 year after diagnosis of gout. The non-colchicine group comprised individuals who had never used colchicine. We excluded gout patients who did not take colchicine within 1 year. This process makes our patient groups more specific to the relation between gout and colchicine. Therefore, we set 1 year after gout diagnosis as our index date. Next, to confirm new-onset pneumonia, we excluded cases diagnosed with pneumonia before the index date in both groups. Then, we matched the two groups based on propensity score in a 1:1 ratio by age, gender, hypertension, chronic liver disease (CLD), chronic kidney disease (CKD), chronic obstructive pulmonary disease (COPD), diabetes mellitus (DM), and gout diagnosis year. After performing propensity score matching, gout patients were precisely distributed into two groups, i.e., with and without colchicine use. Hence, it was possible to observe differences in the rates of new-onset pneumonia between the two groups and to determine factors associated with pneumonia in gout patients taking colchicine.

### Endpoints

Pneumonia was defined as a diagnosis with one or more of the following ICD-9-CM codes: 481, 482, 483, 485, and 486. To ensure the diagnosis, an inpatient or emergency diagnosis of pneumonia was also required for inclusion in the study. To find the relation between colchicine and pneumonia, the diagnosis date of pneumonia had to be at least 1 year after the diagnosis of gout. Patients were followed until pneumonia was diagnosed, withdrawal from the NHI, or until the end of 2013. The abovementioned definitions are for the dependent variables, and the following definitions are for the independent variables. The study period was January 1, 2000, to December 31, 2013. All gout patients were diagnosed with three outpatient or one inpatient (ICD-9-CM = 274) during the period 2000 to 2012.

Colchicine use was defined as a prescription with one of the following National Health Insurance (NHI) codes: A005225100, A0217541G0, A022077100, A022534100, A021754100, A030396100, A0303961G0, A041316100, A0413161G0, A046680100, A048749100, A0487491G0, A054643100, AC54643100, B022246100, N006271100, and N0062711G0 in the study period.

The age of the patients was defined on the index date. For the sex variable, 0 represented females and 1 represented males. We selected the comorbidities of pneumonia. Two outpatient visits or one inpatient diagnosis of comorbidities of pneumonia a year after the index date was required to ensure diagnostic accuracy. The baseline characteristics were age, gender, hypertension (ICD-9-CM = 401-405), CLD (ICD-9-CM = 571), CKD (ICD-9-CM = 585), COPD (ICD-9-CM = 490-492, 494, 496), and DM (ICD-9-CM = 250).We defined that 0 represent patients who had no comorbidities were represented by 0, those who developed a comorbidity were represented by 1. Then, we adjusted for age, gender, hypertension, CLD, CKD, COPD, and DM by applying propensity score matching to eliminate any bias that might confound the identification of differences between gout patients with and without colchicine use.

### Statistical Analysis

The comparison of incidental pneumonia between the colchicine group and non-colchicine group was done by Chi-square test and independent t-test. Kaplan-Meier analysis was applied to evaluate the cumulative incidence of pneumonia in the two groups and the log-rank test was to test whether it was significant. Cox proportional hazard model was used to evaluate the hazard ratio (HR) of pneumonia in relation to colchicine dosage as well as days of colchicine use, after adjustment for potential confounding variables (Austin, 2011). Subgroup analysis by age, gender, hypertension, CLD, CKD, COPD, and DM was performed after applying the Cox proportional hazard model. SPSS version 18.0 (SPSS Inc., Chicago, IL, USA) statistical software was used for the analyses. A p value < 0.05 was considered statistically significant.

## Results

**Figure 1:**
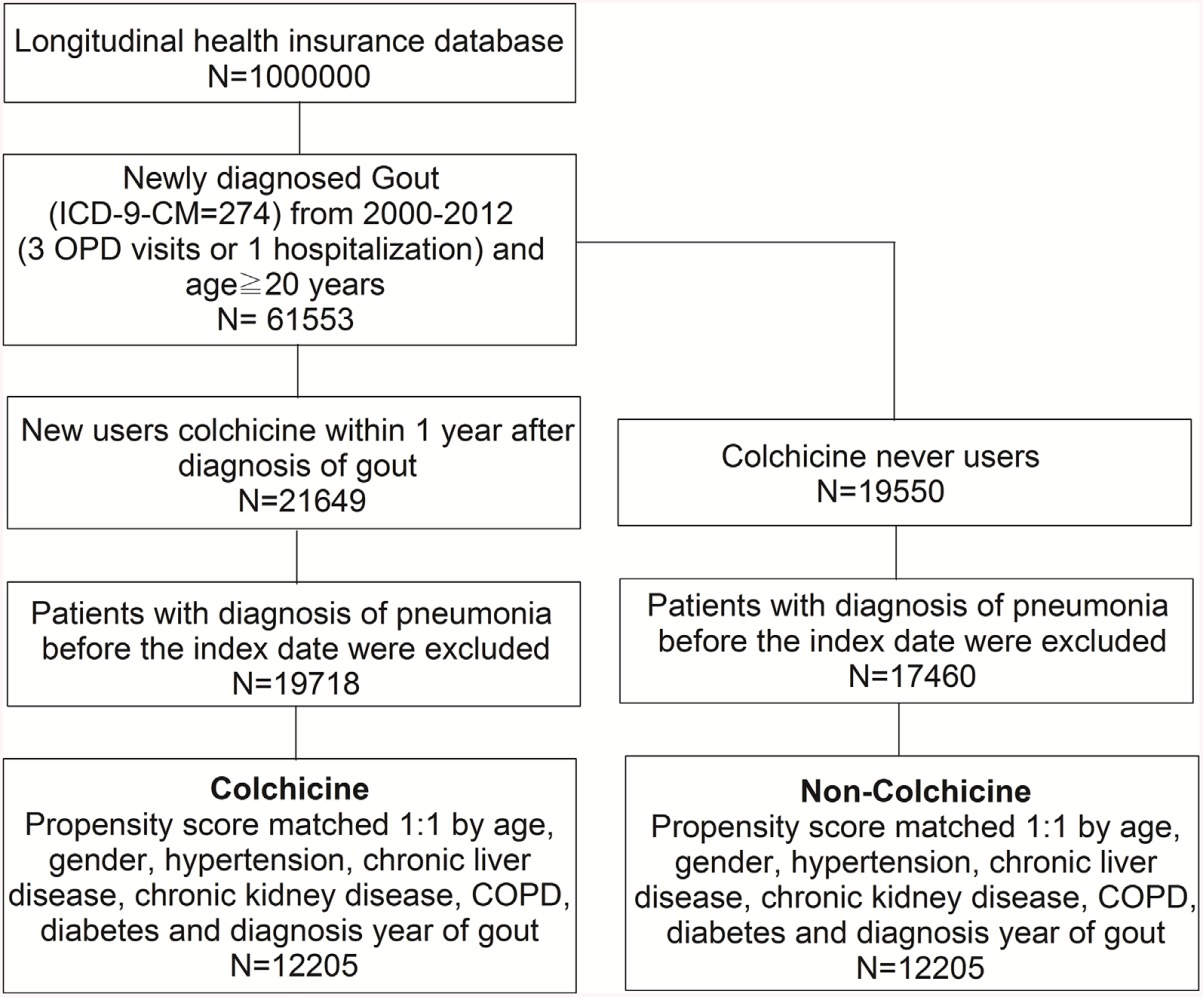
The study population selection protocol.

A total of 24,410 gout patients with and without colchicine use, who had been selected from the NHIRD, were included in the final analysis after propensity score matching. The flowchart of the study population selection protocol is shown in **Figure 1**.

**Table 1** shows that the colchicine group and non-colchicine group both had similar age and gender distributions after propensity score matching. Moreover, we found that males and patients aged 20 to 65 years predominated. The mean ages of the colchicine and non-colchicine groups were 55 (SD = 16) and 54.1 (SD = 15.3) years, respectively. As compared with the non-colchicine group, the colchicine group had a higher prevalence of hypertension at baseline (p < 0.05). The prevalence rates of the other comorbidities, i.e., CLD, CKD, COPD, DM, did not show significant differences between the two groups (p > 0.05).

**Table 1:** Demographic characteristics of gout patients treated with and without colchicine.

	Before PS matched		*p*-value	After PS matched		*p*-value^a^
	Colchicine (N = 19,718), n (%)	Non-Colchicine (N = 17,460), n (%)		Colchicine (N = 12205), n (%)	Non-Colchicine (N = 12205), n (%)	
Age, year			<0.001			**<0.001**
20–65	15,548 (78.9)	12,543 (71.8)		8,560 (70.1)	8,978 (73.6)	
≧65	4,170 (21.1)	4,917 (28.2)		3,645 (29.9)	3,227 (26.4)	
Mean ± SD	49.8 ± 16.6	55.4 ± 14.8	<0.001	55 ± 16	54.1 ± 15.3	**<0.001**
Gender			<0.001			0.108
Female	3,582 (18.2)	7,359 (42.1)		3,582 (29.3)	3,697 (30.3)	
Male	16,136 (81.8)	10,101 (57.9)		8,623 (70.7)	8,508 (69.7)	
Hypertension	5,530 (28)	7,458 (42.7)	<0.001	4,908 (40.2)	4,738 (38.8)	**0.026**
Chronic liver disease	1,860 (9.4)	3,139 (18)	<0.001	1,836 (15)	1,765 (14.5)	0.200
Chronic kidney disease	343 (1.7)	332 (1.9)	0.243	248 (2)	246 (2)	0.928
COPD^b^	943 (4.8)	1057 (6.1)	0.688	751 (6.2)	690 (5.7)	0.098
Diabetes	1,793 (9.1)	3,482 (19.9)	<0.001	1,743 (14.3)	1,835 (15)	0.096

a Bold font represents statistical significance (p< 0.05).

b COPD, chronic obstructive pulmonary disease.

**Figure 2:**
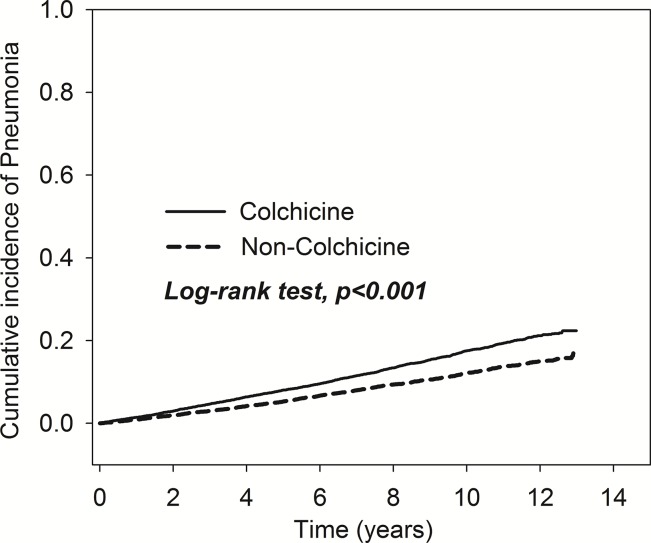
Comparison of cumulative incidences of pneumonia in the patients with and without taking colchicine.

As shown in **Figure 2**, the results of Kaplan-Meier analysis revealed that the cumulative incidence of pneumonia rose over time. The trend can be observed in both the colchicine and non-colchicine groups. Furthermore, the colchicine group showed a higher cumulative incidence of pneumonia than the non-colchicine group (log-rank test, *p* < 0.001).

**Table 2** demonstrates that the overall incidence rates of pneumonia, in the colchicine group and non-colchicine group, were 18.6 and 12.6 per 1,000 person-years, respectively. After adjustment for age, gender, and comorbidities, the colchicine group exhibited a higher risk of pneumonia compared with the non-colchicine group [adjusted HR (aHR), 1.42; 95% confidence interval (CI), 1.32 to 1.53]. The incidence of pneumonia was higher in gout patients 65 years or older than in those aged 20 to 65 years and was higher in males than in females. Using a Cox proportional hazard model, the adjusted HR of pneumonia was not only 4.41-fold higher in gout patients 65 years or older than in those aged 20 to 65 years (95% CI, 4.06–4.80), but was also 1.26-fold higher in males than in females (95% CI, 1.16–1.37). Moreover, the risk of pneumonia was higher in patients with hypertension, CKD, COPD, and DM (*p* < 0.05).

**Table 2:** Analysis of factors affecting pneumonia risk in gout patients treated with and without Colchicine by Cox proportional hazard model.

	No. of Pneumonia event	Observed Person-Years	Incidence Density (Per 1000 Person-Years)	Crude HR (95% CI) ^a^	Adjusted HR^c^ (95% CI) ^a^
Colchicine					
No	1,127	89,638	12.6	1	1
Yes	1,687	90,748	18.6	**1.48 (1.37-1.59)**	**1.42 (1.32-1.53)**
Age					
20-65	1,057	136,667	7.7	1	1
≧65	1,757	43,720	40.2	**5.37 (4.98-5.80**)	**4.41 (4.06-4.80)**
Gender					
Female	828	54,199	15.3	1	1
Male	1,986	126,187	15.7	1.03 (0.95-1.12)	**1.26 (1.16-1.37)**
Hypertension	1,575	64,894	24.3	**2.31 (2.14-2.49)**	**1.33 (1.22-1.44)**
Chronic liver disease	364	28,135	12.9	0.80 (0.71-0.89)	1.01 (0.90-1.13)
Chronic kidney disease	102	2,416	42.2	**2.90 (2.38-3.53)**	**1.75 (1.43-2.13)**
COPD^b^	404	9,566	42.2	**3.02 (2.71-3.35)**	**1.80 (1.61-2.00)**
Diabetes	643	23,187	27.7	**2.04 (1.87-2.23)**	**1.63 (1.49-1.78)**

a Bold font represents statistical significance (p < 0.05).

b COPD, chronic obstructive pulmonary disease.

c Adjusted for age, gender, hypertension, chronic liver disease, chronic kidney disease, COPD and diabetes.

In **Table 3**, the result of the subgroup analysis revealed that the aHRs of the colchicine group compared with the non-colchicine group based on demographic factors and comorbidities. Patients aged 20 to 65 years and 65 years or older in the colchicine group were more likely to develop pneumonia than their respective counterparts in the non-colchicine group. Moreover, with respect to gender, there was a significant difference in the incidence of pneumonia between the colchicine group and the non-colchicine group. In the colchicine group, all of the selected comorbidities were associated with increased risk of pneumonia. Moreover, the greatest magnitude of aHR could be observed in patients with chronic liver disease.

**Table 3:** Subgroup analysis of pneumonia risk in gout patients with and without colchicine treatment by Cox proportional hazard model.

	Colchicine		Non-Colchicine		
	N	No. of Pneumonia	N	No. of Pneumonia	HR^b^ (95% CI)^a^
Age (years)					
20-65	8,560	639	8,978	418	**1.58 (1.39-1.78)**
≧65	3,645	1,048	3,227	709	**1.36 (1.23-1.49)**
Gender					
Female	3,582	505	3,697	323	**1.50 (1.31-1.73)**
Male	8,623	1,182	8,508	804	**1.40 (1.28-1.53)**
Hypertension					
No	7,297	735	7,467	504	**1.46 (1.31-1.64)**
Yes	4,908	952	4,738	623	**1.41 (1.27-1.56)**
Chronic liver disease					
No	10,369	1,456	10,440	994	**1.41 (1.30-1.53)**
Yes	1,836	231	1,765	133	**1.57 (1.27-1.94)**
Chronic kidney disease					
No	11,957	1,629	11,959	1083	**1.42 (1.32-1.53)**
Yes	248	58	246	44	1.37 (0.91-2.08)
p for interaction = 0.869					
COPD					
No	11,454	1,451	11,515	959	**1.43 (1.32-1.55)**
Yes	751	236	690	168	**1.40 (1.14-1.71)**
Diabetes					
No	10,462	1,299	10,370	872	**1.39 (1.28-1.52)**
Yes	1,743	388	1,835	255	**1.53 (1.30-1.79)**

a Bold font represents statistical significance (p < 0.05).

b Adjusted for age, gender, hypertension, chronic liver disease, chronic kidney disease, chronic obstructive pulmonary disease (COPD), and diabetes.

**Table 4** shows the risk of developing pneumonia based on cumulative days of colchicine use. Individuals taking colchicine for less than 8 days, more than 8 days but less than 32 days, and more than 33 days had aHRs of 1.33 (95% CI, 1.20-1.48), 1.45 (95% CI, 1.31-1.60), and 1.47 (95% CI, 1.33-1.62), respectively. That is to say, the risk of developing pneumonia increased with duration of colchicine use. Cumulative dose of colchicine was divided into three groups: less than 9 mg, more than 9 mg but less than 24 mg, and more than 24 mg. The aHRs were 1.38 (95% CI, 1.25-1.53), 1.43 (95% CI, 1.29-1.58), and 1.45 (95% CI, 1.31-1.60), respectively. The risk of pneumonia increased with cumulative dose of colchicine.

**Table 4:** Analysis of pneumonia risk in gout patients based on Colchicine dose and duration using Cox proportional hazard model.

	N	No. of Pneumonia events	Observed Person-Years	Incidence Density (Per 1000 Person-Years)	Crude HR (95% CI)^a^	Adjusted HR^b^ (95% CI)^a^
Cumulative days of Colchicine use						
No	12,205	1,127	89,638	12.6	1	1
<8 days	3,890	481	29,962	16.1	**1.27 (1.15-1.42)**	**1.33 (1.20-1.48)**
8-32 days	4,207	587	31,204	18.8	**1.50 (1.35-1.65)**	**1.45 (1.31-1.60)**
≧33 days	4,108	619	29,583	20.9	**1.66 (1.51-1.84)**	**1.47 (1.33-1.62)**
Cumulative dose of Colchicine use						
None	12,205	1,127	89,638.43	12.6	1	1
Low (<9 mg)	3,916	529	29,599.42	17.9	**1.42 (1.28-1.58)**	**1.38 (1.25-1.53)**
Median (9-24 mg)	4,198	552	31,262.56	17.7	**1.40 (1.27-1.55)**	**1.43 (1.29-1.58)**
High (≧24 mg)	4,091	606	29,886.03	20.3	**1.61 (1.46-1.78)**	**1.45 (1.31-1.60)**

a Bold font represents statistical significance (p < 0.05).

b Adjusted for age, gender, hypertension, chronic liver disease, chronic kidney disease, chronic obstructive pulmonary disease (COPD), and diabetes.

## Discussion

This is the largest real-world study on the risk of pneumonia in gout patients using colchicine. We found that colchicine increased the risk of contracting pneumonia in gout patients. Our results showed a 42% greater hazard of pneumonia in gout patients using colchicine compared with gout patients not using colchicine. The risk of pneumonia risk was higher in older gout patients than in younger gout patients. It was also higher in males than in females, and in people with comorbidity compared with those without comorbidity. In addition, risk of developing pneumonia increased in proportion to colchicine dosage and duration of use.

Spaetgens et al. (2017) investigated infections in gout patients, including risk of pneumonia in colchicine users. Their results showed that patients with gout who used colchicine >31 days had a higher risk of contracting pneumonia. In contrast, gout patients who used colchicine ≦30 days had a lower risk, which was not consistent with our results. However, in the present study, the studied population was far larger than that in the UK study, and therefore, our findings may have more accurately reflected the relationship between colchicine and pneumonia.

The mechanism of colchicine to relieve gout involves the inhibition of microtubule formation (Andreu and Timasheff, 1982; Luduena and Roach, 1991). However, microtubule polymerization is related to many cell functions, such as intracellular vesicle transport, secretion of cytokines and chemokines, cell migration, cell division, and regulation of gene expression (Caviston and Holzbaur, 2006). Disruption of microtubule formation may cause adverse effects. A number of reactions resulting from colchicine usage may explain the increased risk of pneumonia infection in gout. First, NADPH oxidase mediates the production of superoxide anions by neutrophils. The assembly of the NADPH oxidase complex is disrupted by interference of microtubule polymerization. Therefore, by using low doses of colchicine, superoxide produced by neutrophils can be inhibited (Chia et al., 2008). Moreover, superoxide is a factor used by neutrophils to fight against viruses and bacteria (Winterbourn et al., 2016). Thus, if the NADPH oxidase-superoxide system is weakened, there is a greater likelihood of pneumonia infection. Second, by decreasing neutrophil l-selectin expression and changing the distribution of E-selectin on endothelial cells, colchicine can decrease neutrophil recruitment and adhesion to inflamed tissues (Cronstein et al., 1995). Accordingly, as the effectiveness of neutrophils passing through blood vessels has been reduced, they may not be able to reach tissue and fight against bacteria. Third, leukotriene B4 (LTB4) is a mediator of inflammation and a chemo-attractant. LTB4 also takes part in promoting the adhesion and mobility of neutrophils. Colchicine dramatically decreases leukocyte adherence and emigration induced by LTB4 (Asako et al., 1992). Paschke et al. investigated the effect of colchicine on the regulation of cell motility. They postulated that colchicine could modulate the stiffness, elasticity, and viscosity of neutrophils through the reorganization of subcellular compartments (Paschke et al., 2013). Hence, colchicine is thought to affect the motility and deformability of neutrophils in specific places at therapeutic doses. To date, the mechanism of the relationship between colchicine and pneumonia remains unclear, although intriguing recent evidence has shed light on the apparent immuno-suppressive characteristic of colchicine. It is known that the cytochrome P450 (CYP) 3A4 enzyme can metabolize colchicine. Recently, Dalbeth et al. confirmed that combination of colchicine and CYP3A4 inhibitors, such as cyclosporin, tacrolimus, and imidazole, may increase intracellular accumulation of colchicine, which can lead to increased infections (Dalbeth et al., 2014; Stack et al., 2015). The abovementioned findings support our hypothesis that colchicine, an immunosuppressive drug widely used in the treatment of gout, weakens the immune system, rendering the patient prone to pneumonia infection.

This is the first study to investigate the relationship between cumulative doses of colchicine and pneumonia. We postulated that colchicine was a risk factor and applied 1:1 propensity score matching to adjust for demographic factors and comorbidities. A major strength of this study was the use of a nationwide population-based database (NHIRD). The NHIRD allows researchers to readily determine the incidence and correlations of selected factors for virtually the entire population of Taiwan. Also, the retrospective nature of the study design minimized any potential selection bias, reference bias, and participant bias in this population-based study. The analysis of data obtained from the NHIRD for the period 2000 to 2013 in Taiwan revealed that gout patients taking colchicine had a significantly greater risk of developing pneumonia, and there was also a significant dose-dependent effect.

Our study had several limitations. First, information about lifestyle, such as smoking habit and alcohol consumption, are not collected in the claims-based insurance database. To reduce this bias, we adjusted for COPD, which would cover smoking habit, chronic liver disease, which would reflect alcohol consumption, and other comorbidities. Second, there is no genomic variables in this study because the NHIRD does not record related data. Third, all of the diagnoses in the NHIRD were made by physicians using ICD-9-CM codes. Thus, the severity or pathogen of pneumonia was not available in this database. Patients with mild pneumonia may not have been included in our analysis because we selected pneumonia patients diagnosed from emergency visits or admissions as well as gout patients diagnosed with at least three outpatient visits or one admission to ensure that only patients with an accurate diagnosis were selected. Finally, our study design was retrospective so additional prospective studies are needed to elucidate the causal relationship between colchicine and pneumonia. Furthermore, risk factors may vary among different countries, and thus further research is necessary to confirm the association found in this study.

In conclusion, this nationwide population-based cohort study revealed that colchicine use was associated with higher risk of pneumonia in gout patients. Therefore, it is crucial that gout patients taking colchicine be prescribed the minimally effective dosage for as short a duration as possible to minimize the risk of developing pneumonia.

## Data Availability

The raw data supporting the conclusions of this manuscript will be made available by the authors, without undue reservation, to any qualified researcher.

## Author Contributions

T-LT, JC-CW, Y-TW, Y-HK, K-LL, and Y-HW participated in the design of the study. Y-HW was involved in collecting data and producing tables. T-LT, Y-TW, Y-HK, and K-LL produced the initial draft of the manuscript that was further revised by JC-CW and J-YC. All co-authors reviewed and approved the final version of the manuscript.

## Conflict of Interest Statement

The authors declare that the research was conducted in the absence of any commercial or financial relationships that could be construed as a potential conflict of interest.

## Abbreviations

aHR, adjusted HR; CKD, chronic kidney disease; CLD, chronic liver disease; COPD, chronic obstructive pulmonary disease; DM, diabetes mellitus; HR, hazard ratio; ICD-9-CM, The International Statistical Classification of Diseases and Related Health Problems, 9th Revision Clinical Modification; LHIRD 2000, Longitudinal Health Insurance Research Dataset 2000; NHI, National Health Insurance; NHIRD, National Health Insurance Research Database; WHO, World Health Organization.

## References

[B1] Andreu J. M. Timasheff S. N. (1982). Tubulin bound to colchicine forms polymers different from microtubules. Proc. Natl. Acad. Sci. U. S. A. 79 (22), 6753–6756. 10.1073/pnas.79.22.6753 6960347 PMC347211

[B2] Asako H. Kubes P. Baethge B. A. Wolf R. E. Granger D. N. (1992). Colchicine and methotrexate reduce leukocyte adherence and emigration in rat mesenteric venules. Inflammation 16 (1), 45–56. 10.1007/BF00917514 1312060

[B3] Austin P. C. (2011). An introduction to propensity score methods for reducing the effects of confounding in observational studies. Multivariate Behav. Res. 46 (3), 399–424. 10.1080/00273171.2011.568786 21818162 PMC3144483

[B4] Beggs A. E. Reeves D. J. Noel N. S. (2012). Leukopenia associated with long-term colchicine administration. Am. J. Health Syst. Pharm. 69 (24), 2147–2148. 10.2146/ajhp120330 23230037

[B5] Caviston J. P. Holzbaur E. L. (2006). Microtubule motors at the intersection of trafficking and transport. Trends Cell Biol. 16 (10), 530–537. 10.1016/j.tcb.2006.08.002 16938456

[B6] Chen H. H. Perng W. T. Chiou J. Y. Wang Y. H. Huang J. Y. Wei J. C. (2018). Risk of dementia among patients with Sjogren’s syndrome: a nationwide population-based cohort study in Taiwan. Semin. Arthritis Rheum. 48 (5), 895–899. 10.1016/j.semarthrit.2018.06.007 30075989

[B7] Chia E. W. Grainger R. Harper J. L. (2008). Colchicine suppresses neutrophil superoxide production in a murine model of gouty arthritis: a rationale for use of low-dose colchicine. Br. J. Pharmacol. 153 (6), 1288–1295. 10.1038/bjp.2008.20 18264123 PMC2275448

[B8] Cronstein B. N. Molad Y. Reibman J. Balakhane E. Levin R. I. Weissmann G. (1995). Colchicine alters the quantitative and qualitative display of selectins on endothelial cells and neutrophils. J. Clin. Invest. 96 (2), 994–1002. 10.1172/JCI118147 7543498 PMC185287

[B9] Dalbeth N. Lauterio T. J. Wolfe H. R. (2014). Mechanism of action of colchicine in the treatment of gout. Clin. Ther. 36 (10), 1465–1479. 10.1016/j.clinthera.2014.07.017 25151572

[B10] Dixon A. J. Wall G. C. (2001). Probable colchicine-induced neutropenia not related to intentional overdose. Ann. Pharmacother. 35 (2), 192–195. 10.1345/aph.10184 11215839

[B11] Hainer B. L. Matheson E. Wilkes R. T. (2014). Diagnosis, treatment, and prevention of gout. Am. Fam. Physician 90 (12), 831–836. 25591183

[B12] Hsing A. W. Ioannidis J. P. (2015). Nationwide population science: lessons from the taiwan national health insurance research database. JAMA Intern. Med. 175 (9), 1527–1529. 10.1001/jamainternmed.2015.3540 26192815

[B13] Hsu M. H. Yeh Y. T. Chen C. Y. Liu C. H. Liu C. T. (2011). Online detection of potential duplicate medications and changes of physician behavior for outpatients visiting multiple hospitals using national health insurance smart cards in Taiwan. Int. J. Med. Inform. 80 (3), 181–189. 10.1016/j.ijmedinf.2010.11.003 21183402

[B14] Kuo C. F. Grainge M. J. See L. C. Yu K. H. Luo S. F. Zhang W. (2015a). Epidemiology and management of gout in Taiwan: a nationwide population study. Arthritis Res. Ther. 17, 13. 10.1186/s13075-015-0522-8 25612613 PMC4342824

[B15] Kuo C. F. Grainge M. J. Zhang W. Doherty M. (2015b). Global epidemiology of gout: prevalence, incidence and risk factors. Nat. Rev. Rheumatol. 11 (11), 649–662. 10.1038/nrrheum.2015.91 26150127

[B16] Luduena R. F. Roach M. C. (1991). Tubulin sulfhydryl groups as probes and targets for antimitotic and antimicrotubule agents. Pharmacol. Ther. 49 (1–2), 133–152. 10.1016/0163-7258(91)90027-J 1852786

[B17] Paschke S. Weidner A. F. Paust T. Marti O. Beil M. Ben-Chetrit E. (2013). Technical advance: inhibition of neutrophil chemotaxis by colchicine is modulated through viscoelastic properties of subcellular compartments. J. Leukoc. Biol. 94 (5), 1091–1096. 10.1189/jlb.1012510 23901122

[B18] Roddy E. Choi H. K. (2014). Epidemiology of gout. Rheum. Dis. Clin. North Am. 40 (2), 155–175. 10.1016/j.rdc.2014.01.001 24703341 PMC4119792

[B19] Shen Y. Tian Z. Lu D. Huang J. Zhang Z. Li X. (2016). Impact of pneumonia and lung cancer on mortality of women with hypertension. Sci. Rep. 6 (1), 20. 10.1038/s41598-016-0023-2 28442743 PMC5431340

[B20] Smilde B. J. Woudstra L. Fong Hing G. Wouters D. Zeerleder S. Murk J. L. (2016). Colchicine aggravates coxsackievirus B3 infection in mice. Int. J. Cardiol. 216, 58–65. 10.1016/j.ijcard.2016.04.144 27140338

[B21] Spaetgens B. de Vries F. Driessen J. H. M. Leufkens H. G. Souverein P. C. Boonen A. (2017). Risk of infections in patients with gout: a population-based cohort study. Sci. Rep. 7 (1), 1429. 10.1038/s41598-017-01588-5 28469154 PMC5431148

[B22] Stack J. Ryan J. McCarthy G. (2015). Colchicine: new insights to an old drug. Am. J. Ther. 22 (5), e151–e157. 10.1097/01.mjt.0000433937.07244.e1 24100258

[B23] Su V. Y. Yang K. Y. Yang Y. H. Tsai Y. H. Perng D. W. Su W. J. (2018). Use of ICS/LABA combinations or LAMA is associated with a lower risk of acute exacerbation in patients with coexistent COPD and asthma. J. Allergy Clin. Immunol. Pract. 6 (6), 1927–1935.e1923. 10.1016/j.jaip.2018.01.035 29432960

[B24] Winterbourn C. C. Kettle A. J. Hampton M. B. (2016). Reactive oxygen species and neutrophil function. Annu. Rev. Biochem. 85, 765–792. 10.1146/annurev-biochem-060815-014442 27050287

